# Expanded Graphite as a Superior Anion Host Carrying High Output Voltage (4.62 V) and High Energy Density for Lithium Dual-Ion Batteries

**DOI:** 10.3390/mi15111324

**Published:** 2024-10-30

**Authors:** Tejaswi Tanaji Salunkhe, Il Tae Kim

**Affiliations:** Department of Chemical, Biological, and Battery Engineering, Gachon University, Seongnam-si 13120, Gyeonggi-do, Republic of Korea; 201850068@gachon.ac.kr

**Keywords:** lithium dual-ion battery, energy density, high output voltage

## Abstract

The demand for safer, sustainable, and economical energy storage devices has motivated the development of lithium dual-ion batteries (Li_DIBs) for large-scale storage applications. For the Li_DIBs, expanded graphite (EG) cathodes are valuable as anion intercalation host frameworks to fabricate safer and more cost-effective devices. In this study, three different carbon cathode materials, including microwave-treated expanded graphite (MW-EG), ball-milled expanded graphite (BM-EG), and high-temperature-carbonized carbon nanoflakes (CNFs), were developed by different synthesis methods. Li_DIBs were configured by employing 4 M of LiPF_6_ in a dimethyl carbonate electrolyte and MW-EG/BM-EG/CNF as an anion host cathode. After 600 cycles, a Li-MW-EG Li_DIB exhibited a reversible capacity of 66.1 mAh/g with a high Coulombic efficiency of 96.2% at a current rate of 0.05 A/g and an outstanding average energy density of 298.97 Wh/kg (with an output voltage of 4.62 V). The remarkable electrochemical results are associated with (i) moderate structural defects with a very low *I_D_/I_G_* ratio (0.848), (ii) degree of graphitization, which improves the mechanical stability and conductivity, and (iii) large pore volume and pore diameter, easy facilitating the accumulation of PF_6_^−^ ions. The energy density characteristics demonstrate the feasibility of utilizing MW-EG as a promising cathode for energy-related Li_DIB applications.

## 1. Introduction

Despite the energy density, the use of lithium-ion batteries (LIBs) is hindered by challenges due to the scarcity and high cost of transition-metal minerals used in their electrodes, such as Co and Ni. Moreover, the environmental impact and safety concerns confirm the need for alternative energy storage solutions. In recent years, the landscape of energy storage technologies has been shifting toward sustainable and cost-effective storage systems. This transition is driven by the escalating demand for large-scale energy storage [1,2,3,4].

Amid the impending technologies, lithium dual-ion batteries (Li_DIBs) have garnered attention as promising candidates for the next era of storage technologies. In contrast to conventional LIBs, which operate based on a “rocking-chair” mechanism [5,6,7], Li_DIBs are characterized by dual-ion intercalation and operate based on a “bouncing-ball” mechanism. This approach enables intercalation of both anions (e.g., PF_6_^−^, ClO_4_^−^ TFSI^−^, or FSI^−^) and cations (e.g., Li^+^) [8,9,10] into the positive and negative electrodes, respectively. The anion size is moderately larger than the cation size. Its insertion occurs at a higher voltage, which leads to an increase in the resultant operational voltage. Moreover, due to the larger size of the anion, the electrostatic interaction with cathode materials is partial, and thus the charging of the battery is faster than that of traditional single-ion batteries [8,9,11,12]. This adaptability does not enlarge the merits of active materials (cathodes), which suggests potential compensations in relation to sustainability and cost-effectiveness.

The relatively high voltage and high capacity of the Li_DIB structure is principally attributed to its anion insertion mechanism, as explained above. Future progress is determined by the properties and engineering of the cathode active materials. In this regard, for the advancement of Li_DIBs, it is fundamental to research and architect cathode active materials that exhibit mechanical stability at high voltages, surface activation to improve conductivity, and specific degree of crystallization and surface area, which together contribute to superior electrochemical performances [5,11,12]. Specific anions that are dissolved in nonaqueous electrolytes are polyatomic in nature, such as ClO_4_^−^, PF_6_^−^, AsF_6_^−^, TFSI^−^, and FSI^−^ [13,14]. To hold such polyatomic anions in a reversible manner, the configuration of the electrode must have an expansive interstitial space. This suggests that these electrodes should be held together by van der Waals forces in at least one crystallographic direction, which yields a layered structure. According to requirements, graphite could be a better choice due to its distinct layered structure, affectability, and environmental friendliness, and thus has been frequently employed as a cathode active material in Li_DIBs [15,16,17]. The electrochemistry of anion intercalation is determined by the structure, morphology, synthesis method, surface activation, degree of crystallization, and surface area [17,18].

In this study, we synthesized three different carbon cathode materials, microwave-treated expanded graphite (MW-EG), ball-milled expanded graphite (BM-EG), and high-temperature-carbonized carbon nanoflakes (CNFs). We analyzed MW-EG, BM-EG, and CNF by different characterization techniques to evaluate the degree of crystallization, and the degree of randomness by calculating the *I_D_/I_G_* ratio, surface area, and surface morphology. We investigated the effects of different synthesis treatments on the electrochemical performances of Li_DIBs configured by employing an electrolyte of 4 M of LiPF_6_ in dimethyl carbonate (DMC) and MW-EG/BM-EG/CNF anion host cathode. After 600 cycles, Li-MW-EG Li_DIBs exhibited a reversible capacity of 66.1 mAh/g, notable Coulombic efficiency of 96.2% at a current rate of 0.05 A/g, and an impressive average energy density of 298.97 Wh/kg (with an output voltage of 4.62 V). The high performances of the fabricated Li-MW-EG Li_DIBs through electrochemical means can be linked to three key factors: (i) low content of structural defects with a low *I_D_/I_G_* ratio (0.848), (ii) synthesis method that enhances the surface activation without affecting the graphitization, improving the mechanical stability and conductivity, and (iii) sheets with large pore volume and pore diameter, which aid in the accumulation and spontaneous release of PF6^−^ ions with minimal volume expansion. The energy densities show the potential of using MW-EG as a promising cathode for energy-related Li_DIBs applications. The process of PF6^−^ ion interaction and de-intercalation in MW-EG at varying voltage levels is explained through Raman spectroscopy and energy-dispersive spectroscopy (EDS) for elemental analysis.

## 2. Materials and Methods

### 2.1. Expanded Graphite Prepared by Microwave Treatment (MW-EG)

All chemicals utilized in this procedure were of analytical quality and acquired from commercial sources without further purification. Deionized water was used throughout the synthesis. Natural flake graphite (pristine graphite) (Alfa Aesar, Ward Hill, MA, USA, 99.99%, 200 mesh) was dried in a vacuum oven at 70 °C for 24 h. A blend of 100 mL of nitric acid (70% HNO_3_, Daejun Chemicals and Metals, Siheung-si, Gyeonggi-do, South Korea) and sulfuric acid (95.0–98.0% H_2_SO_4_, Sigma-Aldrich, St. Louis, MO, USA) was subsequently prepared in a beaker at a volume ratio of 1:9. A total of 1.0 g of dry original graphite was measured and mixed into the acid solution with constant stirring for 1 h. Afterward, the blend was kept at ambient temperature for 12 h, which enabled the HNO_3_ and H_2_SO_4_ to penetrate the unaltered graphite and create an intercalated graphite (IG). The IG was washed multiple times with deionized water and passed through a polytetrafluoroethylene (PTFE) membrane filter paper until the solution reached a pH of 6.0 to 7.0. It was then subjected to drying in a vacuum oven at a temperature of 70 °C for a duration of 24 h. A specific quantity of IG was then placed into a glass beaker and heated in a microwave oven (WC-A201KW, 2450 MHz, 700 W) for 45 s. Upon microwave treatment, the IG quickly grew with a noticeable burst, resulting in an expanded graphite. The synthesized expanded graphite by this method is referred to as microwave-expanded graphite (MW-EG) [19].

### 2.2. Expanded Graphite Prepared by Ball Milling (BM-EG)

During a typical procedure, 1 g of pure graphite and 8 g of ammonium bicarbonate underwent ball milling in an 80-cm^3^ ZrO_2_ bowl with ZrO_2_ balls with diameters of 3/8 and 3/16 in, with a ball-to-powder ratio of 20:1. The container was spun at a speed of 300 rpm for a duration of 24 h. After milling, the mixture was transferred to a sealed vial and subjected to heating in a vacuum oven at 70 °C for 20 min. Subsequently, it was cooled to room temperature to facilitate proper intercalation of ammonium bicarbonate into the graphite. The intercalated graphite was then subjected to microwave radiation (WC-A201KW, 2.450 MHz, 700 W) for a duration of 60 s. Upon microwave treatment, the graphite precursor quickly reacted and emitted bursts of light, resulting in the expansion of a sheet of graphite. The final product was subjected to annealing at a temperature of 500 °C for 15 min in a tubular furnace to completely remove any leftover ammonium bicarbonate in the air. The synthesized expanded graphite by ball milling is referred to as ball-milled expanded graphite (BW-EG) [20].

### 2.3. Preparation of High-Temperature-Carbonized Carbon Nanoflakes (CNFs)

CNFs were produced through a straightforward one-step calcination process. 10 g of sodium citrate was carbonized at 850 °C for 1 h in an argon atmosphere, with a temperature ramp rate of 5 °C/min. The produced material was washed with warm deionized water (~50 °C) and pure ethanol to remove the template created in the material (sodium carbonate). The cleaned item was dehydrated in a vacuum oven at a temperature of 80 °C for a duration of 12 h, yielding CNFs [21].

### 2.4. Physical Characterization and Electrochemical Measurements

X-ray powder diffraction (XRD; Rigaku D/max 2200 system, Tokyo, Japan) was used to analyze the crystal structures and phase purities of the synthesized materials. The analysis was conducted at the Smart Materials Research Center for IoT at Gachon University, utilizing a monochromatic radiation Cu Kalpha1 = 1.540562 Å. The XRD machine was set to 40 kV and 30 mA. We gathered data in a 2*θ* range of 10° to 60° at a rate of 5°/min. Further structural information was acquired via Raman spectroscopy by utilizing an DM500i (DongWoo) system equipped with a 633-nm laser (Gyeonggi-do, Republic of Korea). The synthesized material’s specific surface area was calculated using the Brunauer–Emmett–Teller (BET) nitrogen adsorption–desorption method (ASAP 2020, Corp., Norcross, GA, USA). Scanning electron microscopy (SEM) was used to analyze the morphology of the material (Hitachi S-4700, Tokyo, Japan).

The cathode material for Li_DIB was fabricated using MW-EG, BM-EG, and CNF. A mixture was prepared with 60 wt% of the prepared carbon, 30 wt% of polyacrylic acid, and 10 wt% of Super P carbon in ethanol as a solvent. The slurry was spread onto an aluminum foil and subsequently dried in a vacuum at 70 °C for 12 h. The EG mass loading on the electrodes ranged from 0.85 to 1.2 mgcm^−2^. A glovebox was used to create a 4-M electrolyte solution with an ultrahigh-purity LiPF_6_ salt and DMC solvent. CR2032 coin cells were constructed inside a glovebox filled with argon, with lithium metal as an anode, the prepared carbon electrode as a positive electrode, and a polyethylene separator. The performance of the electrochemical system was evaluated using a WBCS300 battery testing system (WonATech, Seoul, Republic of Korea), operating in the voltage range of 2−5 V and cycling at a current rate of 0.05 Ag^−1^ for 600 cycles. After the 600 cycles, the rate capability was assessed at current densities of 0.05 to 0.7 Ag^−1^. Changes in the operating voltage of MW-EG, BM-EG, and CNF were examined while cycling at varying rates. An ex-situ Raman analysis was used to investigate the structural changes in MW-EG during charging and discharging at different voltages. The analysis was conducted on the positive electrodes using a DM500i (DongWoo, Gyeonggi-do, Republic of Korea) micro-Raman spectrometer (600 nm) after the 2 initial cycles at low current. An ex-situ EDS analysis was conducted to confirm the intercalation of the PF_6_^−^ anions into BM-EG.

## 3. Results and Discussion

**Figure 1:**
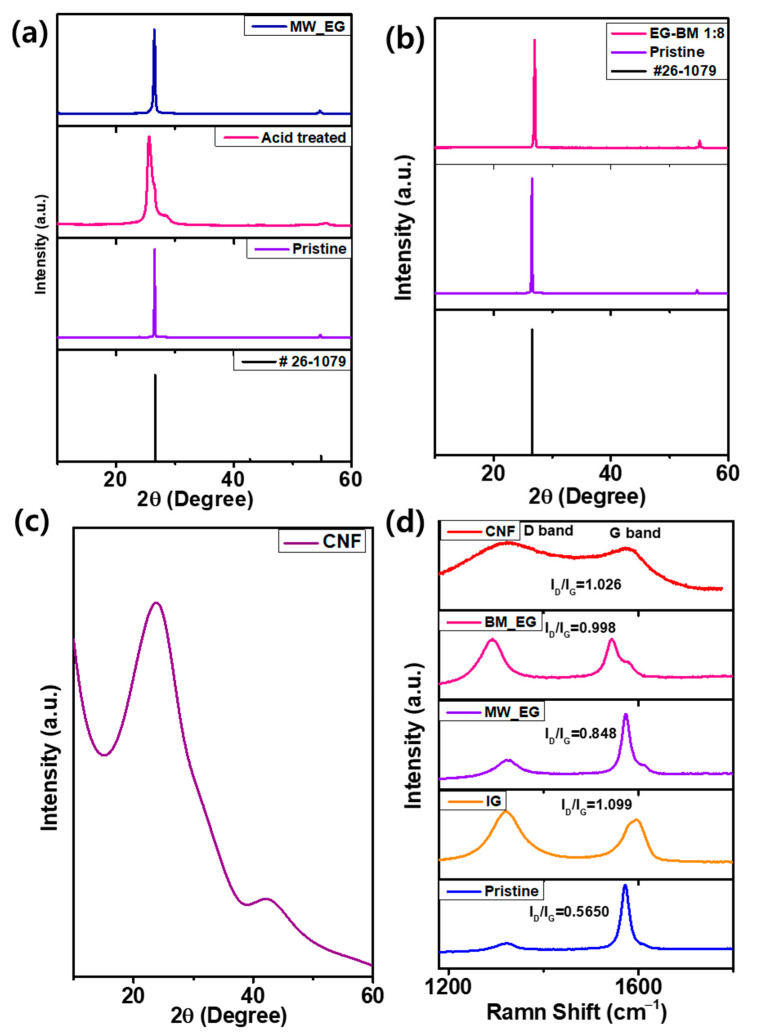
XRD patterns of the (**a**) pristine graphite, acid-treated graphite, MW-EG, (**b**) EG-BM, and (**c**) CNF, and (**d**) pristine, IG, MW-EG, EG-BM, and CNF Raman spectra.

The XRD was carried out to investigate the crystal structures of the as-processed carbons. MW-EG and BM-EG exhibited a strong characteristic peak at 2*θ*~26.5°, corresponding to the (002) plane, which reveals that they preserve the graphitic structure. As shown in Figure 1a, the pristine graphite diffraction peak at 26.6° corresponds to (002), with *d* = 0.34 nm, which indicates a crystalline nature. The XRD pattern for the acid-treated graphite exhibits a broad feeble diffraction peak, shifted to a smaller angle (25.53°) with an increased full width at half-maximum (FWHM) to 1.287. This signifies an increase in *d* spacing and reduction in crystallinity with disorder owing to the acid treatment. Compared to the pristine graphite, MW-EG exhibits a broad peak at ~26.5° (*d* = 0.336 nm). The pattern for MW-EG shows a considerably larger FWHM (0.319) than that of the pristine sample (0.149). The degree of disorder is higher, and the layer of sheets is smaller [1]. The structure of graphite can be described in terms of its degree of graphitization (DG) and crystal perfection (CP). The degree of graphitization refers to the extent to which the carbon atoms are arranged in a perfectly ordered graphite lattice, while crystallinity refers to the size and perfection of the graphite crystals. Anions intercalate more effectively in graphite with a higher degree of graphitization and better crystal perfection because well-ordered graphitic structures provide more accessible pathways for anion intercalation and deintercalation, leading to higher charge/discharge capacities and improved cycling stability [19,20,21].

Based on the model proposed by Maire and Mering, the degree of graphitization can be calculated from the following equation: [22,23]

$$DG\%=\frac{0.3440-d_{002}}{0.3440-0.3354},$$

where 0.3440 nm is the interlayer spacing of the fully non-graphitized carbon, 0.3354 nm is the interlayer spacing of the ideal graphite crystallite, and *d*_002_ is the interlayer spacing derived from the XRD pattern of the studied material.

The calculated *DG*% for MW-EG is 0.6255%, whereas that for the acid-treated graphite is a negative value of 0.4558%, indicating that the acid-treated material does not have carbon atoms arranged in a perfectly ordered graphite lattice, which indicates a highly nongraphitic crystal structure. The XRD pattern in Figure 1b shows that the diffraction peak of BM-EG is broader and weaker than that of the pristine sample, as the BM and ammonium bicarbonate could increase the distortion degree and FWHM by 0.201. However, we did not observe an impurity peak of ammonium bicarbonate, which indicates that it acts as a template. The XDR pattern of CNF shows a characteristic broad 002 peak, corresponding to a larger *d* than that of the above EG (Figure 1c). The calculated *DG*% for CNF was −3.5116%, implying that the material has a nongraphite structure with unordered carbon. The interlayer spacing is a critical factor for ensuring stable electrochemical performance in Li_DIBs [22].

The Raman measurements after the treatment indicate significant amounts of edge deformations and topologic defects, reflected by substantial changes in the intensities of the D band at ~1323 cm^−1^ and G band at ~1572 cm^−1^. The D band corresponds to the presence of defects and disorder in the graphite lattice, while the G band is associated with the in-plane vibration of sp^2^-bonded carbon atoms. As shown in Figure 1d, compared to the pristine carbon, the treated carbon exhibits a significantly increased intensity of the D band. The *I_D_/I_G_* ratios were 0.57, 1.099, 0.848, 0.998, and 1.026 for the pristine, acid-treated MW-EG, BW-EG, and CNF samples, respectively. According to the ratios, the order of distortions is acid-treated structure > CNF > BW-EG > MW-EG > PG. The acid-treated sample manifests functional groups on the surface, reflecting the formation of a more disturbed structure. BW-EG has a higher distortion than MW-EG, likely owing to the long milling time with intercalation of the gaseous template. CNF exhibits a high *I_D_/I_G_* ratio owing to gas generated by the sodium citrate, which leads to an increased concentration of imperfections of the carbon matrix by producing pores and lower-temperature graphitization. The MW-EG carbon has a favorably ordered structure, with a comparatively low content of structural imperfections and upgraded structural reliability [24,25,26].

**Figure 2:**
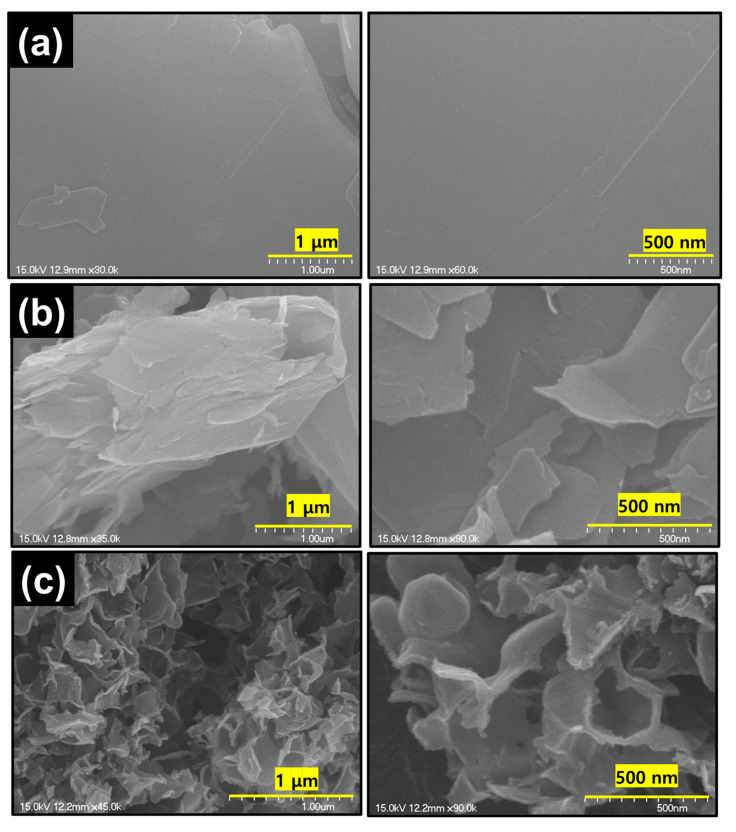
SEM images of the (**a**) pristine graphite, (**b**) BM-EG, and (**c**) CNF at resolutions of 1 µm and 500 nm.

To understand the morphological changes throughout the synthesis process, we carried out an SEM analysis. Figure 2a shows SEM images of the pristine graphite with a layered and stacked structure with long sheets. Figure 2b shows the more disturbed sheet and exfoliated structure of BW-EG. This confirms the effectiveness of the milling and ammonium bicarbonate gaseous bubble template intercalation in the sheets. This approach collectively changes the ordered morphology to an exfoliated morphology of BW-EG. This exfoliated nature of the graphite supports the easy intercalation/deintercalation of the PF_6_^−^ ions in/out of the sheet. The SEM image of the CNF in Figure 2c shows the nanoflake-like structure of the carbon with a very small thickness of the flakes of ~35–50 nm, which provides a short ion diffusion path and fast ion transport.

**Figure 3:**
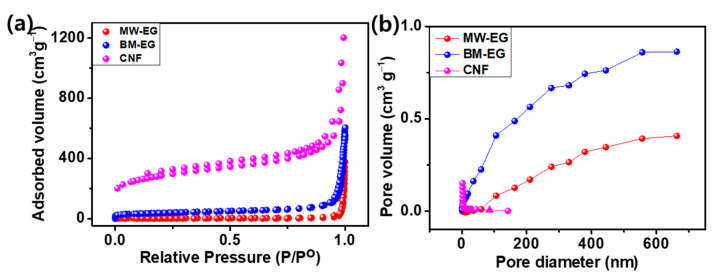
(**a**) Nitrogen desorption–adsorption isotherms and (**b**) corresponding pore size distributions of MW-EG, BM-EG, and CNF.

As the pore structure of the carbon has a key role in the electrochemical performance of Li_DIBs, BET tests were carried out on all carbons to elucidate the effects of the different preparation methods on the surface area of carbon. According to Figure 3, the N_2_ adsorption–desorption isotherms of CNF, BW-EG, and MW-EG show that the specific surface areas are 995.9, 126.7, and 6.66 m^2^g^−1^, respectively. Thus, MW-EG exhibits a relatively very small surface area (19.0 times smaller than that of BW-EG) after the acid and microwave treatments. However, the ammonium bicarbonate interacts with the surface of the carbon, and the simultaneous ball milling effects on the graphite exfoliation result in a considerable increase in the surface area. CNF delivered a 7.8 times larger surface area than that of BW-EG, owing to the precursor used and the carbonization process in the synthesis. The CNF has significantly large surface areas with very small pore diameters and pore volumes compared to BM-EG and MW-EG. The large pore diameter and pore volume of BM-EG and MW-EG provide an appropriate platform for PF_6_^−^ion deposition and transport.

### Electrochemical Analysis

**Figure 4:**
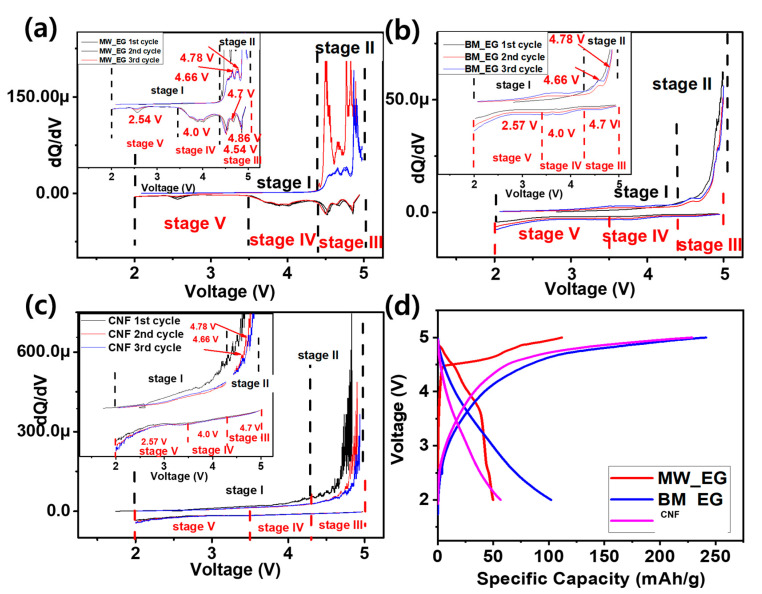
d*Q*/d*V* differential curves of the Li_DIBs with (**a**) Li-MW-EG, (**b**) Li-BM-EG, and (**c**) Li-CNF, and (**d**) initial charge-discharge profile of the Li-MW-EG, Li-BM-EG, and Li-CNF.

The capabilities of the prepared CNF, BW-EG, and MW-EG carbon materials for storage of PF_6_^−^ ions were examined using assembled 2032-type cells with Li metal as an anode and prepared CNF/BW-EG/MW-EG as a positive electrode at a very high concentration (4 M) of the LiPF_6_ electrolyte in DMC. A high concentration of electrolyte is used for a higher availability of PF_6_^−^ ions to improve the energy density of the Li_DIBs. In the case of the Li-carbon Li_DIBs, the anions (PF_6_^−^) in the electrolyte are stored in the cathode during charging, while, during discharging, the stored anions in the electrode are subsequently released back to the electrolyte. This process can be evaluated by a differential capacity plot (DCP) of the Li-carbon Li_DIBs. The initial three DCP peaks of Li-MW-EG, Li-BM-EG, and Li-CNF Li_DIBs are presented in Figure 4a–c, respectively. All Li_DIBs were operated at a current rate of 50 mAg^−1^ in a voltage window adjusted to 2–5 V. The DCPs of all Li-carbon Li_DIBs are divided into five stages according to the peaks. Charging peaks in the potential region of 2–4.3 V (stage I) and 4.3–5.0 V (stage II) correspond to the different intercalation processes of the PF_6_^−^ ions into the carbon sheets. The discharge peaks were in three potential regions of 5–4.3 V (stage III), 4.3–3.4 V (stage IV), and 3.4–2.0 V (stage V), which correspond to various deintercalation phases of PF_6_^−^ ions from the carbon. Very sharp intercalation peaks in stage II are observed at 4.66, 4.78, and 4.89 V, while sharp deintercalation peaks are observed at 4.86, 4.7, 4.54, 4.0, and 2.54 V for the Li-MW-EG Li_DIB. However, the intercalation and deintercalation peaks were less intense for the Li-BM-EG and Li-CNF Li_DIBs, likely owing to the surface activation, high degree of graphitization, and optimal surface area of MW-EG. The DCP curves of Li-MW-EG, Li-BM-EG, and Li-CNF Li_DIBs show the overall reliability of the electrochemical reaction in the Li_DIBs, excluding the first activation step. After the first activation process, the peak position shifted owing to the high voltage intercalation of the PF_6_^−^ ion in the lattice of the carbon, creating lattice expansion and possible deformation of the carbon matrix. This initial expansion modifies the intercalation sites available for anions, leading to a different peak position in subsequent cycles. as Additionally, electrolyte decomposition at ~5 V leads to the formation of a (CSEI) layer, an irreversible process that affects the intercalation/deintercalation mechanism of subsequent cycles. Initial cycles often introduce strain in the carbon layers due to the large PF_6_^−^ ions. In subsequent cycles, the electrode may undergo relaxation or slight reordering, resulting in a stabilized intercalation pathway and a different peak position from the second cycle. Some intercalation sites may become partially irreversible due to structural rearrangements during the first cycle. The DCP shows specific electrochemical reaction routes in the Li_DIBs, in line with previous reports [16,17,27,28,29].

The distinctive galvanostatic plots of the first cycles of all Li_DIBs were consistent with the DCP results. Anion intercalation capacities of 111.7, 241.5, and 228.8 mAhg^−1^ were obtained for the Li-MW-EG, Li-BM-EG, and Li-CNF Li_DIBs, with Coulombic efficiencies (CEs) of 44.6%, 42.3%, and 24.7%, respectively. The low CE could be attributed to electrolyte decomposition on the electrode interface at a very low current density of 0.05 Ag^−1^. The 100% deintercalation of the PF_6_^−^ ions from the electrolyte cannot be realized during discharging, which might stockpile in the cathode as a residual form. This is referred to as activation of the electrode [10,27]. Remarkably, the Li-MW-EG and Li-BM-EG cathodes exhibited better initial CEs than the Li-CNF Li_DIB. The MW-EG and BM-EG carbon structures have adequate crystallinity degrees, which improve the deintercalation of the anions. CNF has a very low DG, which largely affects the sequential and easy deintercalation of PF_6_^−^ ions. The working voltages of the Li_DIBs vary with the nature of the carbon cathode. These values affect the overall energy densities of the Li_DIBs and are crucial factors in the selection of the carbons as cathodes for Li_DIBs. The working voltages of the initial cycles were 4.50, 3.63, and 3.46 V for the Li-MW-EG, Li-BM-EG, and Li-CNF Li_DIBs, respectively. The Li-MW-EG Li_DIB had a very high working voltage, compared to the other Li_DIBs.

**Figure 5:**
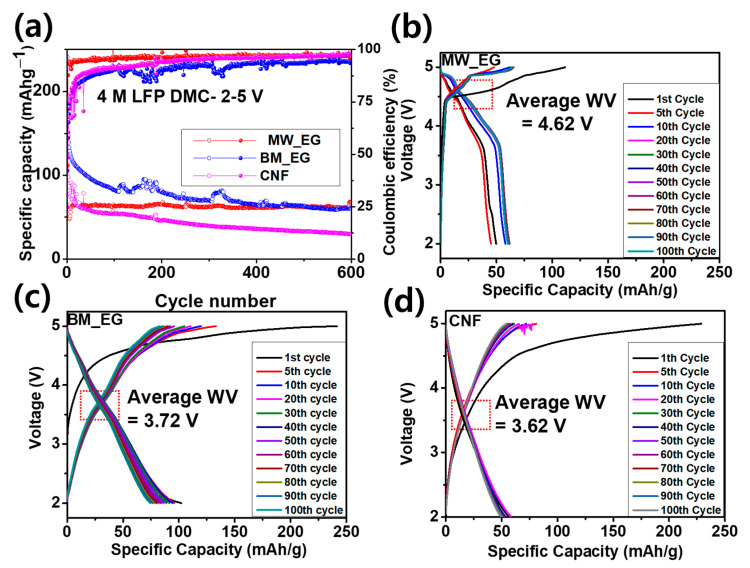
(**a**) Long-term cycling results of the Li-MW-EG, Li-BM-EG, and Li-CNF Li_DIBs at a current density of 0.05 A/g. Charge–discharge profiles of the initial 1st, 5th, 10th, 20th, 30th, 40th, 50th, 60th, 70th, 80th, 90th, and 100th cycles of the (**b**) Li-MW-EG, (**c**) Li-BM-EG, and (**d**) Li-CNF Li_DIBs with their delivered average working voltages.

The charge–discharge profiles of the long cycling performances of the Li-MW-EG, Li-BM-EG, and Li-CNF Li_DIBs are presented in Figure 5b–d. The initial charge–discharge capacities and corresponding CEs are presented in Table 1. All Li_DIBs delivered very low initial CE owing to DMC revealing the largest polarization at a high voltage with a low current, the accumulation of PF_6_^−^ ions inside the EG cathode as residual form, and a large amount of loss in the initial cycle due to the formation of cathode solid electrolyte interphase (CSEI) at high voltages, resulting in reduced restorage capability and marginal CE values. Nevertheless, the CE subsequently increased with the cycling to 96.1%, 88.3%, and 92.4% with charge capacities of 62.5, 86.9, and 51.2 mAhg^−1^ for the Li-MW-EG, Li-BM-EG, and Li-CNF Li_DIBs, respectively, after 150 cycles. They further increased to 96.2%, 94.3%, and 97.6% after 600 cycles with charge capacities of 68.1, 60.4, and 29.8 mAhg^−1^ for the Li-MW-EG, Li-BM-EG, and Li-CNF Li_DIBs, respectively. After three initial cycles, the CE results of all three batteries are very promising, even at a very low current density of 0.05 Ag^−1^. The retentions of charge capacity were 60.95%, 25.0%, and 13.02% for the Li-MW-EG, Li-BM-EG, and Li-CNF Li_DIBs, respectively, after 600 cycles with respect to the second cycle. Among the three batteries, the Li-MW-EG Li_DIB delivered a very stable long-cycling performance with a high charge capacity of 62.5 mAhg^−1^ with a retention of 60.95% after 600 cycles. These results were attributed to moderate structural defects with a very low *I_D_/I_G_* ratio (0.848). The synthesis method helps activate the surface, improves the conductivity, and provides higher mechanical stability, a specific degree of crystallization of MW-EG, and sheets with a small surface area, large pore volume, and large pore diameter (Figure 3b). The smaller surface area suppressed the side reaction at a high voltage. The large pore diameter and pore volume facilitated the stage of accumulation of PF_6_^−^ ions and spontaneous release with a very small volume expansion of MW-EG. In contrast, CNF delivered an extremely low-capacity retention of 13.02%, with a very low capacity of only 29.8 mAhg^−1^, owing to carbon sheets with a large *d* spacing and very poor DG, with a highly distorted structure with a very high *I_D_/I_G_* ratio (1.026 according to the Raman analysis). Such structure could be easily destroyed during the initial cycling because of its lower mechanical stability, which results in very poor cycling stability. In addition, the extremely large surface area led to a very thick cathode solid electrolyte interphase (CSEI) layer on the surface and side reactions, which, in turn, led to the unavailability of anions to provide capacity. BM-EG delivered a very high initial capacity (241.5 mAh/g) because of its moderate surface area, which is conducive to the intercalation–deintercalation kinetics of the PF_6_^−^ ions throughout the cycling. However, it did not exhibit good retention owing to its very low mechanical stability (because of its higher *I_D_/I_G_*). After several cycles, the cathode suffered from an uncontrollable volume expansion. As a result, the cathode detached from the current collector and reduced the electrochemical performance [10,12,15,30,31]. Figure 5a illustrates that BM-EG exhibits unstable cycling performance, likely due to the effects of the ball milling process with ABC, which introduces defects and significantly increases surface area (126.7 m^2^ g^−1^, approximately 19 times higher than MW-EG). While a larger surface area with high pore volume and diameter (Figure 3a,b) can enhance ion accessibility, it also accelerates side reactions with the electrolyte, reducing cycling stability. Additionally, the highly defective structure of BM-EG (*I_D_*/*I_G_* ratio of 0.998) is less effective in controlling volume expansion over prolonged cycling, potentially leading to detachment from the current collector, which impairs electrical conductivity and further destabilizes cycling performance. Milling-induced structural changes may also introduce new intercalation mechanisms and result in greater irreversible capacity loss. The defective structure of BM-EG could cause uneven ion distribution during PF_6_^−^ intercalation, leading to inhomogeneity and contributing to cycling instability. Remarkably, the Li-MW-EG, Li-BM-EG, and Li-CNF Li_DIBs maintained average working voltages as high as ~4.62, ~3.72, and ~3.62 V (Figure 5c and Figure 5d), respectively, which yielded the superior energy densities of the Li_DIBs. The calculations show that the Li-MW-EG, Li-BM-EG, and Li-CNF Li_DIBs have energy densities of 298.97, 261.92, and 143.79 Whkg^−1^ after 600 cycles, respectively. Notably, Li-MW-EG and Li-BM-EG delivered higher energy densities of the Li−G Li_DIBs than those of reported Li_DIBs [12,17,30].

**Figure 6:**
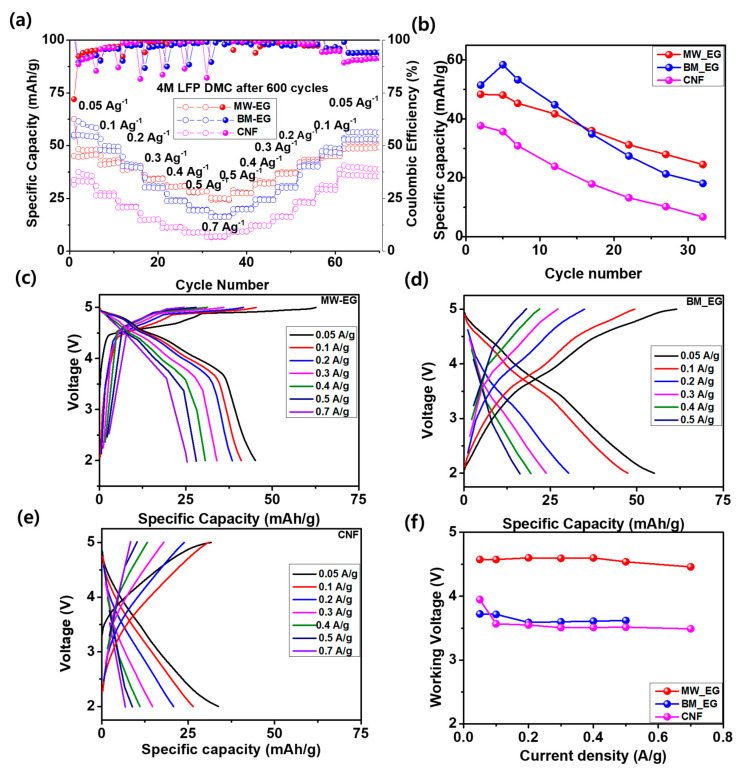
(**a**) Rate capability test on MW-EG, BM-EG, and CNS after cycling at different current rates. (**b**) Specific capacity relationships of MW-EG, BM-EG, and CNS. Charge–discharge profiles of the (**c**) MW-EC, (**d**) BM-EG, and (**e**) CNS. (**f**) Comparison of discharge voltages at different current densities of MW-EG, BM-EG, and CNS Li_DIBs.

Several cycling rate capability tests were carried out on the Li-MW-EG, Li-BM-EG, and Li-CNF Li_DIBs after 600 cycles at altered current densities from 0.05 to 0.7 A/g, with a voltage window of 2.0–5.0 V, as shown in Figure 6a. Among all samples, the Li-MW-EG Li_DIB delivered rate performances of 48.4, 45.3, 41.7, 36.02, 31.2, 27.9, and 24.5 mAh/g at 0.05, 0.01, 0.2 0.3, 0.4, 0.5, and 0.7 A/g, respectively. The current densities were applied again in reversed order, 0.5, 0.4, 0.3, 0.2, 0.1, and 0.05 A/g. The structure delivered a superior regain capacity of 107.3% with respect to the second cycle. Notably, the retention capacities of all electrodes were ~100% with respect to the discharge capacity of the second cycle, as shown in Table 2. Therefore, a high retention capacity with a high CE is beneficial for the application of EG prepared by MW treatment as a promising cathode material for Li_DIBs. Figure 6b compares the charge capacities at different current rates. MW-EG delivered higher capacities at high current densities with regains > 100% retention at 0.05 A/g owing to its moderate pore volume and pore diameter, good DG, and lower *I_D_/I_G_* ratio, which help maintain its mechanical stability at a high current rate. Figure 6c–e shows the working voltages at the different current rates for all Li_DIBs. The middle discharge voltages of all Li-Li_DIBs did not decrease even at a very high current rate of 0.7 A/g, as shown in Figure 6f. However, the working voltages exhibit large differences according to the nature of the used carbon. MW-EG delivered a very high value of 4.56 V, BW-EG exhibited a value of 3.64 V, and CNF exhibited a very low working voltage of 3.58 V. MW-EG provided the highest working voltage of 4.56 V, which is a very high value compared to numerous reported carbon cathodes for Li_DIBs. As a result, MW-EG is a good choice owing to its high capacity, long cycling stability, high ratability, and ability to deliver a very high energy density considering the high working voltage.

**Figure 7:**
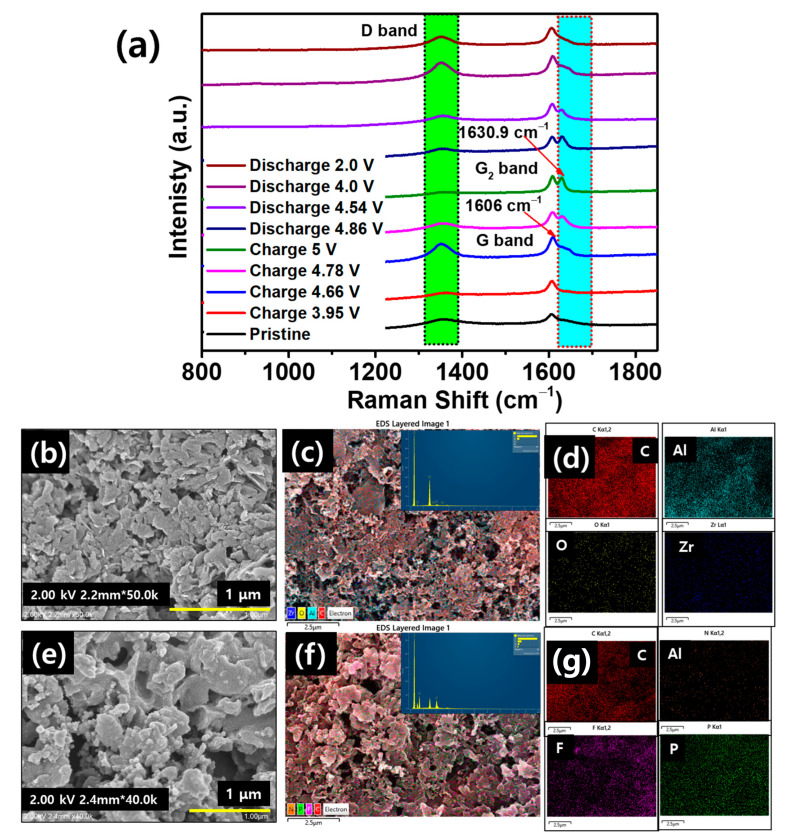
(**a**) Ex-situ Raman analysis of the MW-EG cathode at the pristine state and in the preliminary cycling at various charge–discharge voltages. SEM images of the (**b**) pristine state and (**e**) after full charging of the cathode. (**c**,**d**) and (**f**,**g**) EDS maps of the pristine cathode and cathode after full charging at 5 V, and corresponding spectra, respectively.

The intercalation and deintercalation of PF_6_^−^ ions inside and out of the cathode during different charge–discharge processes were confirmed by an ex-situ Raman analysis of the MW-EG cathode. Further analysis was carried out by ex-situ EDS after full charging with comparison to the pristine EDS. Figure 7a shows that the pristine MW-EG exhibits a sharp peak (G band) at 1606 cm^−1^. Throughout the charging, this peak gradually transformed into a wide shoulder, divided into a dual peak, and a new peak appeared at 1630.9 cm^−1^, attributed to the G_2_ band at 5.0 V. The intensity ratio of *I_G_*_2_/*I_G_* increased with an increase in the charging voltage, as shown in Table 3, from 0.85 at 3.95 V to 0.86 at 4.66 V, 0.93 at 4.78 V, and 0.97 at 5 V. This originates from the sequential intercalation of PF_6_^−^ ions in MW-EG during charging. During discharging, the G_2_ band intensity decreases with the voltage. The *I_G_*_2_/*I_G_* ratio decreased from 1.0 at 4.86 V to 0.89 at 4.54 V, 0.85 at 4.0 V, and 0.83 at 2.0 V. At a full discharge voltage of 2.0 V, only the G band at 1606 cm^−1^ is observed sharply. This implies that PF_6_^−^ ions deintercalated from MW-EG into the electrolyte [27,32]. Moreover, the D band (1350 cm^−1^) slowly decreases with increasing charging voltage and reappears with discharging. These results were further supported by the ex-situ EDS and SEM analyses of the fully charged and pristine cathodes. The SEM image of the pristine BM-EG reveals the smooth surface (Figure 7b), while, after full charging, the BM-EG cathode exhibits an expanded surface, which could be attributed to the intercalation of PF_6_^−^ (Figure 7e). The EDS results of the pristine state (Figure 7c,d) show C, O, Al, and Zr. Al originates from the current collector, while Zr could be attributed to the milling process. The fully charged BM-EG exhibited F and P elements, which could be attributed to the presence of PF_6_^−^ ions in the course of charging [10].

## 4. Conclusions

The nature of the carbon as an anion host for Li_DIBs had a crucial impact on the overall energy density. Three types of carbon materials were designed using microwave, ball milling, and carbonization methods. The anion intercalation capacities of the first cycles were 111.7, 241.5, and 228.8 mAhg^−1^ with retentions of 60.95%, 25.0%, and 13.02% for the Li-MW-EG, Li-BM-EG, and Li-CNF Li_DIBs, respectively, after 600 cycles. The Li-MW-EG Li_DIB delivered a very stable long-cycling performance with a high charge capacity of 62.5 mAhg^−1^ after 600 cycles. Notably, the Li-MW-EG, Li-BM-EG, and Li-CNF Li_DIBs maintained average working voltages as high as ~4.62, ~3.72, and ~3.62 V, with superior energy densities of 298.97, 261.92, and 143.79 Wh kg^−1^, respectively, after 600 cycles. The method of synthesis of carbon strongly affected the DG, *I_D_/I_G_* ratio, pore volume, and pore diameter, which determined the mechanical stability and conductivity, and promoted the electrochemical kinetics of PF_6_^−^ ions. With its excellent performance, Li-MW-EG is the best choice to obtain a high energy density and high output voltage of Li_DIBs.

## Figures and Tables

**Table 1:** Cycling results of Li-MW-EG, Li-BM-EG, and Li-CNF Li_DIBs at different cycle numbers.

4-M DMC Li_DIBs	1st Charge Capacity (mAh/g)	1st Discharge Capacity (mAh/g)	1st Cycle CE (%)	150th-Cycle CE (%)	600th Charge Capacity (mAh/g)	CE (%) at 600	Retention at 600th Cycle wrt 2nd Cycle	Average Working Voltage (V)	Average Energy Density (Wh/kg)
MW-EG	111.7	49.76	44.6	96.1	68.1	96.2	60.95	~4.62	298.97
BM-EG	241.5	102.044	42.26	88.3	60.4	94.3	25.01	~3.72	261.9
CNF	228.8	56.57	24.73	92.4	29.8	97.6	13.02	~3.62	143.8

**Table 2:** Rate capability results at different current densities and regain capacities at 0.05 A/g for the MW-EG, BM-EG, and CNF Li_DIBs.

	0.05 A/g (2nd)	0.1 A/g	0.2 A/g	0.3 A/g	0.4 A/g	0.5 A/g	0.7 A/g	0.05 A/g	Regain Capacity (%) at 0.05 A/g with Respect to the 2nd Cycle
MW-EG capacity (mAh/g)	48.35	45.3	41.7	36.02	31.2	27.94	24.5	51.87	107.3
BW-EG capacity (mAh/g)	51.5	53.3	44.8	34.9	27.4	21.3	18.1	56.3	96.4
CNS capacity (mAh/g)	37.7	30.9	23.9	17.9	13.2	10.2	6.7	40.1	112.3

**Table 3:** The intensity ratio (*I_G_*_2_/*I_G_*) of the ex-situ Raman results at different charge/discharge states.

	Pristine	Charge 3.95 V	Charge 4.66 V	Charge 4.78 V	Charge 5.0 V	Discharge 4.86 V	Discharge 4.45 V	Discharge 4.0 V	Discharge 2.0 V
*I_G_*	35,925.4	38,332.85	45,890.7	40,002.8	40,257.4	42,951.1	40,591.4	45,951.4	40,305.1
*I_G_*_2_	30,000	32,477.7	39,271.3	37,251.3	39,096.3	43,308.3	36,440.2	39,170.1	33,593.1
*I_G_*_2_/*I_G_*	0.84	0.85	0.86	0.93	0.97	1.0	0.898	0.85	0.83

## Data Availability

All materials, data, and associated protocols contained in this manuscript can be made available to readers upon request.
